# Determination of Specific Losses in the Limbs of an Epstein Frame Using a Three Epstein Frame Methodology Applied to Grain Oriented Electrical Steels

**DOI:** 10.3390/s16060826

**Published:** 2016-06-04

**Authors:** Guillaume Parent, Rémi Penin, Jean-Philippe Lecointe, Jean-François Brudny, Thierry Belgrand

**Affiliations:** 1 Laboratoire Systèmes Electrotechniques et Environnement (LSEE), University of Artois, EA 4025, F-62400 Béthune, France; guillaume.parent@univ-artois.fr (G.P.); remi.penin@gmail.com (R.P.); jfrancois.brudny@univ-artois.fr (J.-F.B.); 2 Thyssenkrupp Electrical Steel, F-62330 Isbergues, France; thierry.belgrand@thyssenkrupp.com

**Keywords:** electrical steel, Epstein frame, specific losses, grain oriented

## Abstract

An experimental method to characterize the magnetic properties of Grain Oriented Electrical Steel in the rolling direction is proposed in this paper. It relies on the use of three 25 cm Epstein frames combined to generate three test-frames of different lengths. This enables the identification of the effective specific losses of the electrical steel when magnetization is applied along the rolling direction. As a consequence, it evidences the deviation of the loss figures obtained using the standardised Epstein test. The difference in losses is explained by the fact that the described method gives “only” the losses attached to the straight parts. The concept of the magnetic path length as defined by the standard is discussed.

## 1. Introduction

The Epstein frame is a standardized magnetic circuit regularly used to qualify the properties of electrical steel grades [1]. Some works [2,3,4,5,6] are still devoted to identifying the exactness of the method. A weak point is linked to the use of a magnetic path length which has been set to be equal to 0.94 m [5]. The definition of the mean path length for the field circulation results from a comparison of this magnetic circuit with toroidal circuits made of the same steel grades. Since then, the metallurgic improvement of the steel grades raises the question of the validity of this standardized mean path length. It has been shown that the magnetic path length depends on several parameters attached to the electrical constraints imposed to the magnetic circuit (peak flux density, frequency and waveform of the excitation [3]) as well as the permeability and the anisotropy of the material.

The tremendous improvements in the electrical steel properties during the years may not be without influence on the effective path length. This is especially the case for the Grain Oriented Electrical Steel (GOES) because of the high values of the permeabilities in the Rolling Direction (RD) and of the differences between the RD and the Transverse Direction (TD) properties. Indeed, the anisotropy ratio can reach values up to 20,000.

Several papers have focused on such aspects and they also have proposed the use of various devices based on modified Epstein frames. The oldest one [7] describes a method based on four frames. More recently, Marketos *et al.* used two Epstein frames [3,4]: the standardized 25 cm frame and a shorter one (17.5 cm). Calculating the loss difference enables to access the specific losses [3] but it requires, up to now, us to lay the strong hypothesis that the flux is assumed uniform inside the straight parts of the frame. The authors conclude that taking a mean path length equal to 0.94 m can lead to an underestimation of transformer core building factors. In particular, they show that the higher the permeability, the longer the mean path length. Then, the impacts of the frequency and of the magnetization waveform are studied in [4]. Wang *et al.* [6] have used three square shape Epstein frames of difference sizes to calculate the mean path length. Their limb lengths are equal to 17.5 cm, 20 cm and 25 cm respectively. The mean path lengths for frequencies between 50 Hz and 200 Hz and for different sampling angles (0 ${}^{\circ }$, 55 ${}^{\circ }$ and 90 ${}^{\circ }$ to the RD) are calculated. The authors identify different areas: the overlap area and zones of the straight flux parts called uniform zone and impact zone respectively.

The presented paper also focuses on both the determination of the mean path length and the specific loss, using modified Epstein frames. This is the extension of [8], which relates to a characterization of GOES with two conventional 25 cm Epstein frames. It differentiates from the aforementioned works by the following aspects:

- the modified Epstein frames have longer limbs than the standard frame. That is a crucial point for the experimental approach because steel of high performance is analyzed and, in this case, grains have a cm like size. It is thus more convenient to extend the limb length instead of shorten it, as it has been done up to now,
- the use of three frames instead of two allows to draw more reliable characteristics concerning the influence of the frame size variations,
- only the length of a pair of parallel limbs has a varying value. The two others are kept constant. This feature is determinant to strengthen the hypothesis of the uniformity of the flux in the area close to the corners. It will make it possible to justify that differences on the losses measured in the different frames enable to find the real characteristics of the measured GOES grade.

Various high permeability GOES grades of nominal thickness 0.35 mm, 0.30 mm and 0.23 mm have been tested (Table 1).

## 2. The Reasons for Using Three Different Frame Sizes

### 2.1. Magnetic Circuit Structure

**Figure 1:**
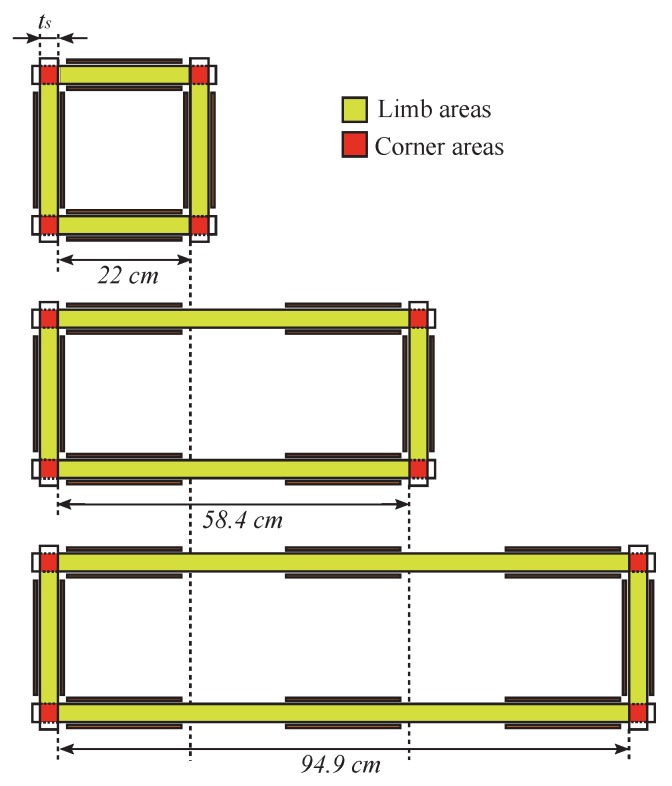
Schematic plan view of three frames. (**Top**) Short (standard Epstein frame); (**Middle**) Medium and (**Down**) Long.

The three Epstein frame method described in this paper relies on experimental measurements. Three different magnetic circuits shown in Figure 1 are built with standardised Epstein frames combined together to create three magnetic circuits differing from each other by the length of the same couple of parallel limbs. Each magnetic circuit of different length is composed of the same grade of steel and is filled with the same number of laminations. They are respectively called Short, Medium and Long frames. The so called Short circuit is a standard Epstein frame whereas the Medium and Long circuits are made respectively from the association in series of two and three Epstein frames. The internal lengths are 22 cm, 58.4 cm and 94.9 cm respectively. Each limbs is composed of $n_{s}=7$ GOES laminations. All samples have been constituted from the same metallurgical batch. All strips were in neighbouring situations from a slit roll. All samples were in the as slit state and they did not undergo stress relief annealing. Figure 1 also shows the corner and limb areas considered in the whole paper.

### 2.2. Experimental Method Description

**Figure 2:**
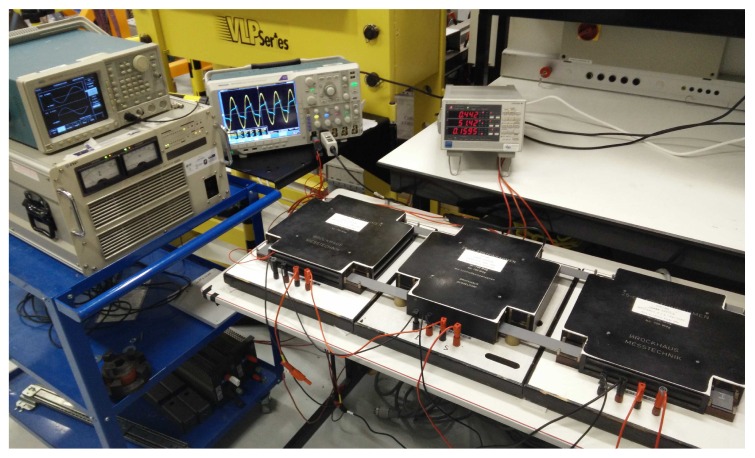
Experimental test bench with the long frame.

The experimental setup is shown in Figure 2 where the long frame is presented together with the measuring equipment. The iron losses $P_{iron}$ are obtained for each frame with a digital wattmeter YOKOGAWA WT203 from the primary current and the secondary voltage. The peak flux density $\hat{b}$ is calculated from the peak secondary voltage $\hat{v_{2}}$ measured across a secondary coil with $N_{2}=175$ turns wound around each Epstein frame limb whose length does not vary. $S=n_{s}t_{s}w_{s}$ is the iron cross section of the limb, where $n_{s}$, $t_{s}$ and $w_{s}$ are the number of sheets per limb, the iron thickness and the width of the sheets, respectively.

(1) $$\hat{b}=\frac{\hat{v_{2}}}{2\pi fN_{2}S}$$

### 2.3. First Experimental Results: Measurement and Magnetic Property Characterizations

**Figure 3:**
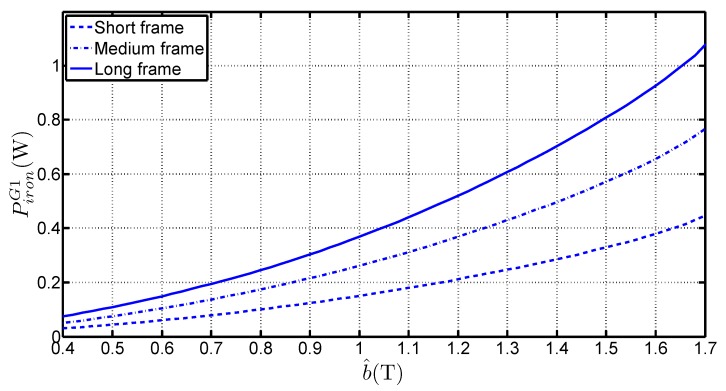
Variations of $P_{iron}$ with $\hat{b}$ for the three frames and G1 steel.

**Figure 4:**
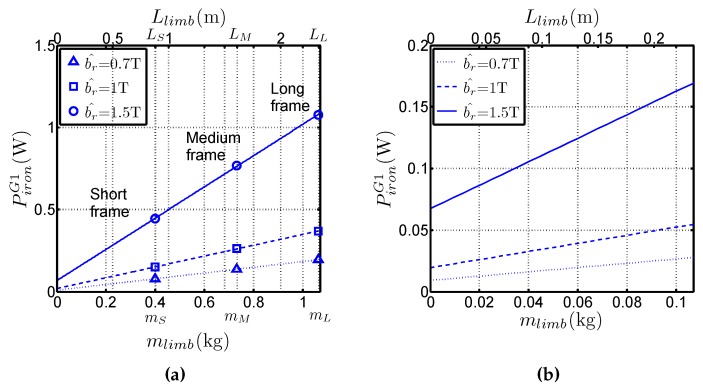
(**a**) Variations of $P_{iron}$ with $m_{limb}$ for $\hat{b}=0.7\;T$, $\hat{b}=1\;T$ and $\hat{b}=1.5\;T$ for G1 steel (**b**) Zoom near the abscissa 0.

Let us denote the following quantities: $P_{iron}$ stands for the total iron losses, $m_{limb}$ is the total limb mass, *i.e.*, excluding the mass of the corners and $L_{limb}$ is the total limb length, *i.e.*, excluding the corners. The experimentally measured iron losses are shown in Figure 3 for the three frames and for G1 grade. The obtained variations are classical with an increase of the losses with the size of the frames and with the flux density. Another representation is shown in Figure 4a which presents the variations of $P_{iron}$ with $m_{limb}$ (bottom horizontal axis) and with $L_{limb}$ (upper horizontal axis) for three values of $\hat{b}$: 0.7 T, 1 T and 1.5 T. The results show a linear trend which enables writing Equation (2), where the slope $p_{iron,l}=\Delta P_{iron}/\Delta m_{limb}$ corresponds to the specific iron losses. The nonzero intercept denoted $P_{iron,0}$ which corresponds to the losses in the corners, differs with $\hat{b}$, as shown in Figure 4b, which presents a zoom of Figure 4a near the abcissa 0. Therefore, it can easily be written:

(2) $$P_{iron}=p_{iron,l}\;m_{limb}+P_{iron,0}$$

The following three characteristics can be extracted from the variation of the losses with the sample mass:

- the slopes of straight lines in Figure 4a give access to $p_{iron,l}$ (which are the real specific losses of the material) without considering the mean path length in the frame, but only with the total limb mass (or with the length increase of the limbs). That constitutes a real advantage of the method,
- $P_{iron,0}$ depends on the mean path length in the corners and it changes with the considered grade and flux density,
- the variations of $P_{iron}$ are linear with $m_{limb}$, even though the medium and long frames are asymmetrical. It constitutes an original approach because it proves that the method is suitable for separating corner losses and limb losses. That confirms the basic hypothesis which considers that the flux distribution is the same near the corners, regardless the frame geometry for a sufficiently long limb. This hypothesis is valid because the short frame dimensions are much bigger than the corner size. Simulations of magnetic behaviour of the laminations [2,9,10] performed with Finite Element software shows that this hypothesis is consistent especially for the GOES because the magnetic polarisation tends to lay along the RD as far as possible in the corner before it changes direction towards adjacent lamination.

### 2.4. Influence of the Frame and Sample Structure

**Figure 5:**
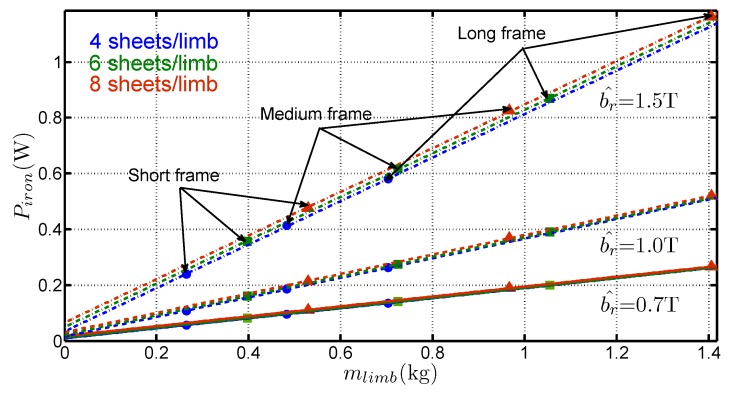
Variations of $P_{iron}$ with $m_{limb}$ for frame equipped of four, six and eight samples per limb (G4).

In order to be sure that the method is robust and accurate, additional tests have been done by modifying the number of samples in the frame. The number of strips per limb have been changed for G4 samples: Figure 5 shows the variations of $P_{iron}$ with $\hat{b}$ for frames made of four, six and eight sheets per limb and for three flux density values. It shows that the losses vary linearly with the increase in limb iron cross section. The fact that the lines are parallel means that $p_{iron,l}$ does not change regardless the limb thickness; this strengthens the validity of the method.

### 2.5. Discussion about the Measurement Uncertainty

As the paper is based on experimental results, the question of the measurement errors arises. Indeed, the method uses the slope determined by three points at a given flux density value. As a results, an error on those three points may lead to a non-negligible error on the determination of $P_{iron,0}$. Nevertheless, in this study, the $P_{iron}$ maximum measurement error is less than 0.5 %. Moreover, Figure 5 shows that the slopes determined for several number of strips are the same. That proves that the measurement are repeatable even if the geometry of the frame has been modified and that the measurement error does not affect the method.

## 3. Experimental Results for Different GO Steel Grades

**Figure 6:**
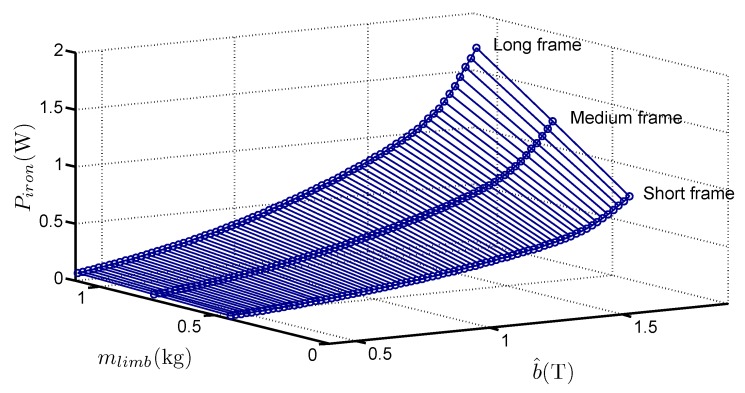
Variations of $P_{iron}$ with the mass increase of the limbs for G1 grade.

The method has been applied to four grades of GOES. For example, Figure 6 shows the variations of $P_{iron}$ with $\hat{b}$ and $m_{limb}$ for the G1 grade.

### 3.1. Specific Loss Determinaton—Comparison with the Epstein Frame

**Figure 7:**
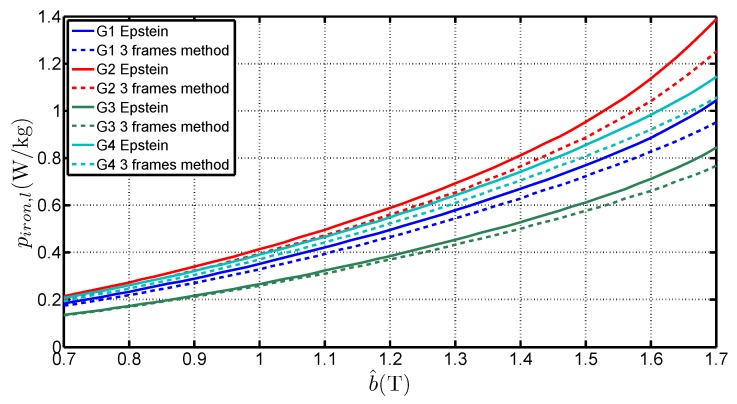
Variations of $p_{iron,l}$ with $\hat{b}$ for four steel grades, measured with the Epstein frame and obtained with the three-frame method.

Applying the described method, $p_{iron,l}$ has been calculated. Figure 7 shows the variations with $\hat{b}$ for the four steel grades, measured with the Epstein frame and obtained with the three-frame method. It puts in evidence that the Epstein frame, applying the IEC 60404-2 standard, leads to an overestimation of the specific losses of about 6%. This tendency, which is already known for GOES [3], is contrary to the results obtained with Non-Oriented Steel [11]. Indeed, the double overlap joint leads to a cross section twice higher in the corners than in the limbs, which corresponds to an average flux density half the one in the limbs. With GOES, the flux tends to magnetize the strips along the RD as long as possible, even in the corners, before passing from a lamination to another adjacent strip. The great advantage of the method is to estimate the specific iron limb losses directly and without the corner effects.

### 3.2. Recalculation of the Epstein Equivalent Mean Path Length

The $L_{e}$ mean path length can be calculated for the Epstein frame (Short frame) through Equation (3) [1,3] where $P_{iron}^{short}$, *l* and $m_{tot}$ are the iron losses of the short frame, the length of a strip (0.305 m) and the total mass of the laminations respectively.

(3) $$L_{e}=4l\frac{P_{iron}^{short}}{p_{iron,l}\;m_{tot}}$$

**Figure 8:**
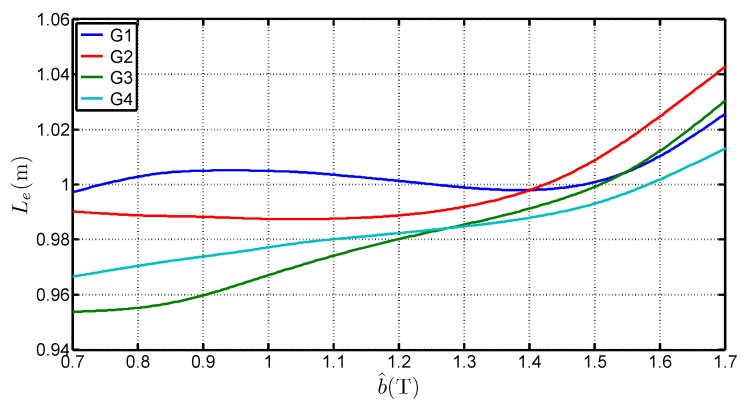
Variations of $L_{e}$ with $\hat{b}$ for four steel grades.

Figure 8 presents the variations of $L_{e}$ with $\hat{b}$ for the four steel grades and it shows that:

- $L_{e}$ is higher than the standardized length. It can even be superior to 1 m which is the geometrical average length of the Epstein frame. The average of the determined values for each grades, considering a $\hat{b}$ range of [0.7 T;1.7 T] is 1.0038 m, 0.9983 m, 0.9817 m and 0.9842 m for G1, G2, G3 and G4 respectively. That shows that the 0.94 m length is too low and not representative of the iron losses,
- $L_{e}$ depends on the peak value of the flux density. The deviation to the standard value is over 6% between 0.7 T and 1.7 T. This expresses the fact that in the corner joints the flux establishes in twice the limb cross section. The local reluctance of the magnetic circuit is thus very different from the one in the limb and influences the overall estimation for the whole magnetic circuit. It also shows that, whatever the grade, this equivalent mean path length tends to increase with the flux density peak value. This variation is similar to the curves obtained by Marketos in [4] for a high performance GOES magnetized with an excitation frequency of 50 Hz,
- $L_{e}$ depends on several parameters. Indeed, $L_{e}$ is influenced by the peak flux density value, the steel grade but also the anisotropy ratio. The latter influences the flux distribution in the corners and may explain the differences on $L_{e}$.

### 3.3. Losses at the Corner Joints

**Figure 9:**
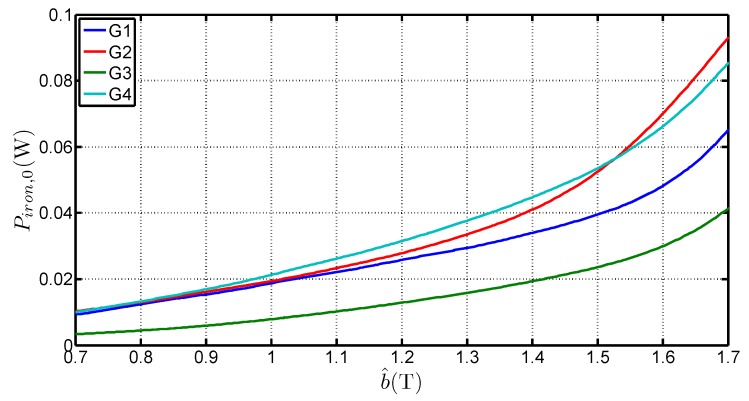
Variations of $P_{iron,0}$ with $\hat{b}$ for four steel grades and obtained with the three-frames method.

**Figure 10:**
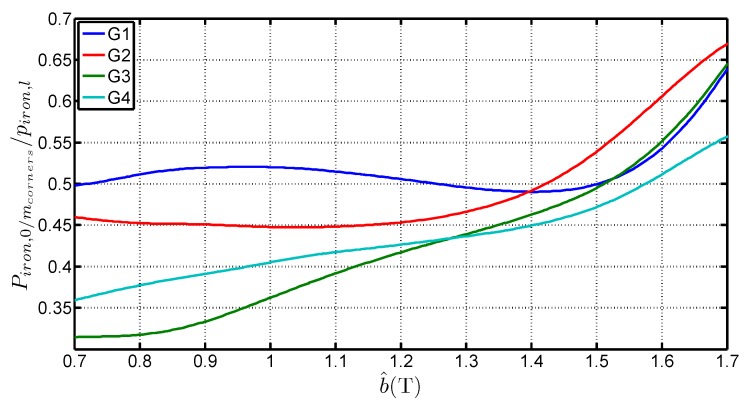
Variations of the ratio between the specific losses in the corners and in the limbs with $\hat{b}$ for 4 steel grades.

Figure 9 gives the iron losses in the four corners, with $\hat{b}$, for the four steel grades. The losses at the corner joints correspond to the intersection of the loss variation trend with the vertical axis. The results vary significantly with the steel grades. For $\hat{b}=1.7\;T$, the corners losses are equal to 15%, 16.5%, 15.7% and 13% of the short frame total losses, respectively for G1, G2, G3 and G4. Figure 10 compares the variations of the specific losses in the corner and in the limbs: $\frac{MMP_{iron,0}\;/\;m_{corners}}{p_{iron,l}}$. Considering the induction level of the limb as the reference it shows that the specific losses in the corners are lower than in the limbs. The variations in Figure 10 show that the specific losses in the corners increase faster with $\hat{b}$ than the specific losses in the limbs. For example at 0.5 T the ratio between corner and limb specific losses is around 1.7 for G2 and T for G1 and at 1 T the ratio is more than 2.5 for each grade.

### 3.4. Equivalent Mean Path Length in the Corner Joints

**Figure 11:**
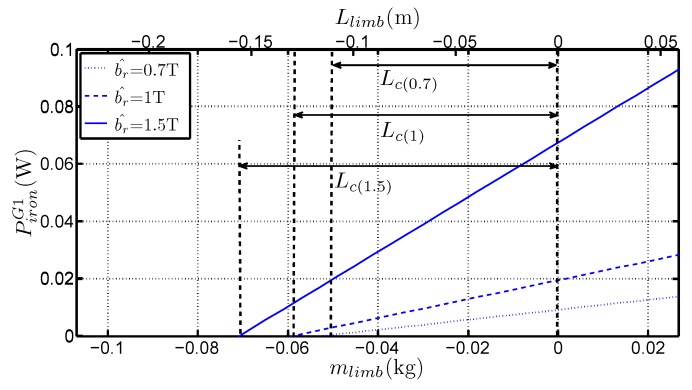
Variations of $P_{iron}$ with $L_{limb}$ for $\hat{b}=0.7\;T$, $\hat{b}=1\;T$ and $\hat{b}=1.5\;T$ for G1 grade.

Starting from the graphs used to calculate the specific losses, the corner losses have been identified by the intersection of the linear trend with the vertical axis. If this line is extended as to reach the point for which the losses are zero, the absolute value (in meters) which is given by the intersection with the horizontal axis is equivalent to a magnetic path length inside the corner joints. Doing such extrapolation led to identify that those values are dependent upon the induction level (see Figure 11 for G1 grade) and grade.

**Figure 12:**
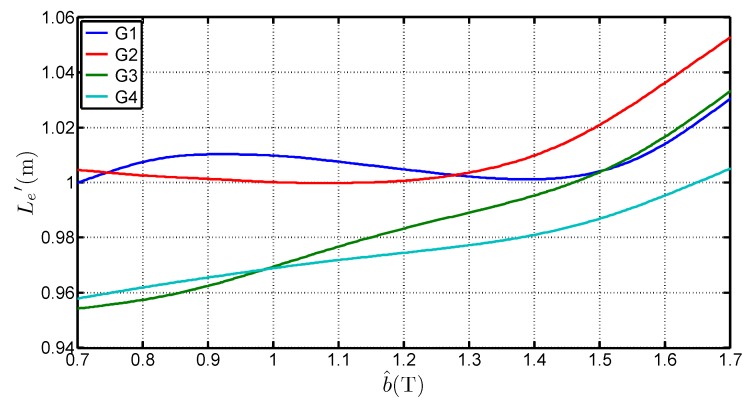
Variations of $L_{e}^{\prime }$ with $\hat{b}$ for four steel grades.

**Figure 13:**
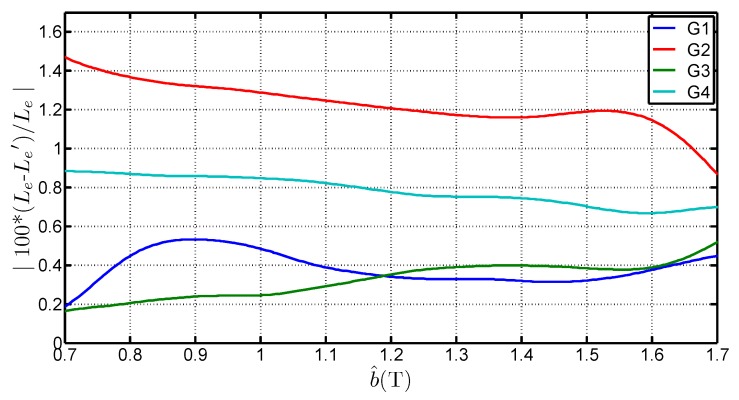
Comparison of $L_{e}^{\prime }$ and $L_{e}$ with $\hat{b}$ for four steel grades.

The flux in the limbs can be reasonably supposed to be homogeneously distributed, even near the corner areas because of the GOES high anisotropy ratio which forces the field to follow the rolling direction. We then sum the equivalent mean path length found in the corners, *i.e.*, $L_{C}$ in Figure 11, with the inter-corner region of the so called Small frame (4×0.22m). It defines a new length noted $L_{e}^{\prime }$ so that : $L_{e}^{\prime }=L_{C}+0.88$. Figure 12 shows the variation of this quantity with $\hat{b}$. The variation is similar to the one in Figure 8. Figure 13 expresses the relative variation of $L_{e}^{\prime }$ and $L_{e}$. On average, the correspondence is less than 1.5 whatever the grades and $\hat{b}$. This raises the question about the meaning of the $L_{e}$ length, which may be assimilated to a mean path of the flux in the corner joint. Considering the Ampere’s law, the sum of the mean path length of the corner and the length of the inter corner area should not correspond to the average path length because a weighting taking into account the magnetizing current would occur into this relationship. This correspondence must, however, be further studied. A complementary work taking into account the peak magnetizing field evolution should be involved.

## 4. Conclusions

The presented method with three frames has three advantages:

- it gives access to the magnetic steel properties without considering the magnetic path length. That constitutes the main advantage because no supposition concerning the mean path length has to be done,
- it makes it possible to separate the corner iron losses and the leg iron losses,
- it shows that the mean path length of the magnetic flux depends on the steel grade and characteristics, but also on the flux density level.

The paper differs from other papers which generally consider two frames of different lengths. Indeed, the chosen frame dimensions and the fact of using three frames make it possible to check that the iron losses are effectively proportional to the frame dimensions and they allow to show that the modification of the limb length does not influence the magnetic flux distribution in and near the corners if the frame dimensions are bigger than the corner dimensions.

## Figures and Tables

**Table 1:** Characteristics of the analyzed electrical steel.

IEC Grade	Grade Name	Iron Losses at 1.5 T	Iron Losses at 1.7 T
	(W · kg${}^{-}$${}^{1}$)	(W · kg${}^{-}$${}^{1}$)	
M105-30P	G1	0.77	1.03
M140-30S	G2	0.95	1.39
M085-23P	G3	0.61	0.84
M135-35P	G4	0.93	1.26
